# The effect of brain metastasis location on clinical outcomes: A review of the literature

**DOI:** 10.1093/noajnl/vdz017

**Published:** 2019-09-13

**Authors:** Pragnan Kancharla, Alexander Ivanov, Susie Chan, Hani Ashamalla, Raymond Y Huang, Ted K Yanagihara

**Affiliations:** 1 Department of Medicine, New York Presbyterian Brooklyn Methodist Hospital, Brooklyn, New York; 2 Department of Radiation Oncology, New York Presbyterian Brooklyn Methodist Hospital, Brooklyn, New York; 3 Department of Radiology, Dana-Farber/Brigham and Women’s Cancer Center, Harvard Medical School, Boston, Massachusetts; 4 Department of Radiation Oncology, University of North Carolina, Chapel Hill, North Carolina

**Keywords:** brain metastases, location, nomogram, prognosis, survival

## Abstract

It is common clinical practice to consider the location of a brain metastasis when making decisions regarding local therapies and, in some scenarios, estimating clinical outcomes, such as local disease control and patient survival. However, the location of a brain metastasis is not included in any validated prognostic nomogram and it is unclear if this is due to a lack of a relationship or a lack of support from published data. We performed a comprehensive review of the literature focusing on studies that have investigated a relationship between brain metastasis location and clinical outcomes, including patient survival. The vast majority of reports anatomically categorized brain metastases as supratentorial or infratentorial whereas some reports also considered other subdivisions of the brain, including different lobes or with particular areas defined as eloquent cortex. Results were variable across studies, with some finding a relationship between metastasis location and survival, but the majority finding either no relationship or a weak correlation that was not significant in the context of multivariable analysis. Here, we highlight the key findings and limitations of many studies, including how neurosurgical resection might influence the relative importance of metastasis location and in what ways future analyses may improve anatomical categorization and resection status.

**Importance of the Study**

The study highlights the key findings and limitations of many studies showing the importance of metastasis location and in what ways future analyses may improve anatomical categorization, prognostic grading and deciding treatment options.

**Key points**

1. The location of a brain metastasis may affect prognosis.
2. The literature informing the relationship between location and clinical outcomes is reviewed.
3. Study limitations impede our understanding of how metastasis location informs clinical practice.

Metastatic brain tumors are the most common intracranial neoplasm in adults with approximately 200 000 cases per year in the United States.^1^ Local therapies for Brain metastases (BMs) include surgery, stereotactic radiosurgery (SRS), whole-brain radiation therapy (WBRT), or some combination of these. Prognosis may be assessed with several validated prognostic tools^2^ that have identified a wide range in the survival estimate based on patient and tumor characteristics. For example, the recent Disease-Specific Graded Prognostic Assessment (DS-GPA) for breast cancer found a median overall survival (OS) of 9.4 months for all patients, but a median OS of 2.6 months and 28.8 months for the lowest and highest prognostic categories, respectively.^3^ A similarly wide range was seen in BM patients with non-small cell lung cancer (NSCLC) primary histology, where the median OS was 9.2 months for non-adenocarcinoma, 15.2 months for adenocarcinoma, and nearly 4 years for adenocarcinoma with the most favorable prognostic score.^4^ Some of the key predictors of survival include performance status, age, extent of extracranial disease, number of BMs, and the histology and molecular features of the primary tumor.^2^

BM location is an important factor when considering local therapies, such as the feasibility and risks of surgery, role for stereotactic radiation, and the radiation dose and fractionation schedule (Fig. 1). However, it is not known if BM location is independently associated with clinical outcomes, including survival and local control (LC), and this factor is not included in widely used prognostic nomograms.^2^ Here, we report results on a comprehensive literature search of studies that assess prognostic variables in patients with BMs that also coded the anatomical location of individual lesions to understand what role this factor might have in determining prognosis.

**Figure 1.**
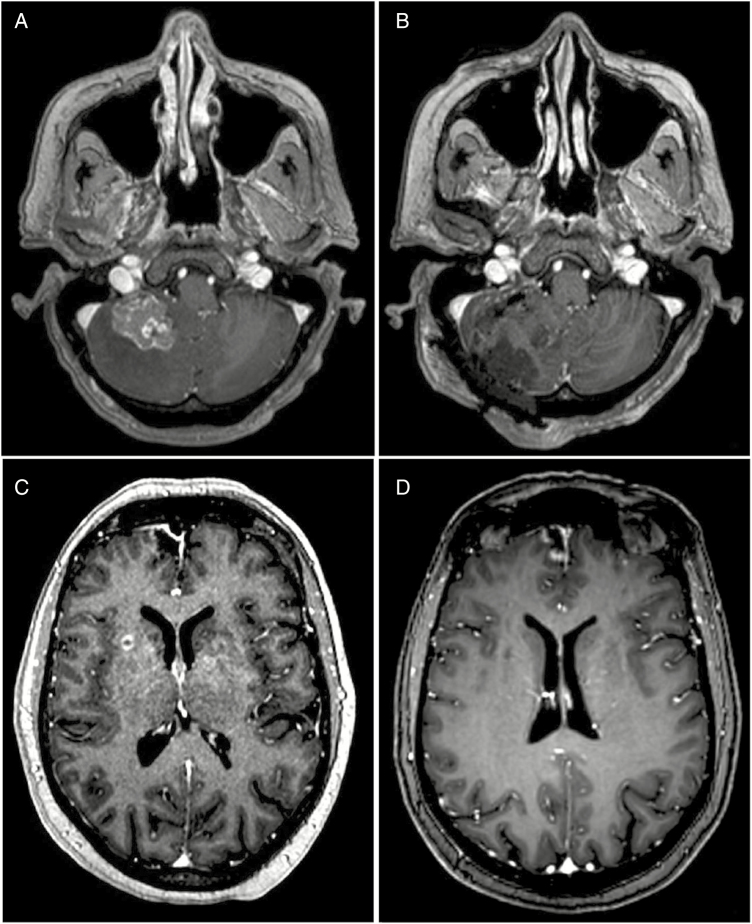
The location of a BMs may influence management decisions. A metastasis from a primary colon cancer in the cerebellum measuring up to 3 cm (**A**). Posterior fossa lesions are often symptomatic and definitive SRS may produce or worsen symptoms related to local edema. Suboccipital craniotomy with stereotactic localization of the tumor resulted in a gross total resection (**B**) that was then managed with SRS to the postoperative bed. A metastatic focus in the right putamen measuring up to 0.7 cm in a patient with breast cancer (**C**). Small lesions in a deep location present an operative challenge and the patient was managed with definitive SRS with complete resolution of the BM after 6 weeks (**D**). BM, brain metastases; SRS, stereotactic radiosurgery.

## Materials and Methods

### Literature Search

The National Library of Medicine (PubMed/MEDLINE) database was used to perform an initial literature search and the Google Scholar (www.scholar.google.com) search engine was used as an additional supplement. The references of identified articles were then reviewed for additional citations to be included. PubMed/MEDLINE searches were up to date and concluded as of May 1, 2019, and Google Scholar searches were up to date and concluded as of May 5, 2019. The database and search engine queries were not restrictive to year of publication; therefore, they include any articles from their inception to the date of query.

The MEDLINE database was queried (Supplementary Table 1) using a series of controlled vocabulary and Medical Subject Headings terms, such as Thesaurus Terms/Subheadings: “Brain Neoplasms” and various text words, for example: [(“brainstem” OR “brain stem” OR “thalamus” OR “frontal lobe” OR “parietal lobe” OR “eloquent” etc.) AND (“metastasis” OR “metastases” OR “secondary” etc.)]. This revealed 665 potential articles. The search was then repeated using the non-MEDLINE (ie, new articles not yet indexed) database, which identified an additional 199 results. These results were then supplemented with an additional PubMed query using the “Best Match” feature, which is more inclusive than the default “Most Recent” feature, and the generic search term “brain metastasis location,” which identified another 953 results. The 1817 results from the PubMed/MEDLINE database were then broadened using the Google Scholar search engine with two additional searches. The first was performed with the term “brain metastasis location” and this identified over 350 000 items and the first 200 hits were reviewed. The cutoff of using the first 200 hits was based on a recently published analysis of systematic literature reviews where Google Scholar is being used as a source.^5^ Beyond 200 hits, there are more redundant and erroneous entries and this cutoff is considered adequate when other sources are being used with at least reasonable yield. An additional Google Scholar query was performed using the search string: “brain metastases prognosis supratentorial OR infratentorial OR thalamus OR brainstem OR lobe -glioblastoma -astrocytoma -glioma” and this identified 34 800 items of which the first 200 were reviewed. Therefore, a total of 2217 journal articles were reviewed individually by two of the investigators (P.K. and T.K.Y.) for inclusion in the final analysis. Supplementary Table 2 summarizes the results of these database queries. To be included in this comprehensive review, a study had to provide some clinical outcome measure (eg, survival, disease progression, treatment toxicity) in patients with BMs where the anatomical locations of the lesions were reported. After excluding duplicate search entries, literature reviews, case reports, non-English language articles, and studies that only included patients with one particular BM location (typically articles specifically evaluating brainstem lesions, unless the article subdivided the brainstem into anatomical subunits or compared to another anatomic location), a total of 126 manuscripts were included.

### End Point Selection

Primary outcome varied among the studies with OS, mean survival, median survival, and LC being the common measures. In addition, each of these outcome measures was reported at various timepoints. Few studies evaluated the association of BM location with the risk of leptomeningeal disease (LMD) or complications. End points that were recorded for each study, where reported, included the following: OS (mean, median, 6-month, 1-year, 2-year, hazard ratio [HR]), LC (median, 6-month, 1-year, relative risk [RR], hazard ratio), HR for LMD, and the odds ratio for complications (neurologic, regional, systemic). Where reported, *P* values for all comparisons relating to BM location were recorded as well as the number of patients in all comparison groups.

## Results

The analysis interval for the studies included in our review was between the years 1944 and 2018. There were several studies focused only on one primary histology, such as NSCLC (*n* = 14, 11.1%) and colorectal cancer (12, 9.5%), but half of them were of multiple histologies (*n* = 63, 50%). The diagnosis, clinical, and neuroimaging follow-up for the studies was using either one or combination of imaging and surgical techniques. Magnetic resonance imaging was the most commonly used imaging modality for BMs although some studies, especially the older entries, did use either computed tomography scanning (*n* = 15, 11.9%) or surgical findings (*n* = 7, 5.6%) alone for tumor localization. Single institution reports accounted for 82.5% (*n* = 104) of studies. The size of the study cohort varied from 15 to 779 patients, with a mean and median of 150.7 (SD = 152.1) and 98, respectively.

Studies used a variety of methods to describe the anatomical location of BMs with the most common being supratentorial versus infratentorial (*n* = 63, 50%). Classification by lobes of the brain were (*n* = 24, 19%) the next most common followed by some other distinction (*n* = 18, 14.3%), such as eloquent versus non-eloquent or brainstem versus cerebellum.

Of the 126 studies that met inclusion criteria and passed all exclusion criteria, 56 (44.4%) did not provide sufficient statistical data or report numerical quantification of their findings in regard to lesion location to allow comparisons to other published literature. For example, some studies included lesion location in a univariable analysis and state that there was no significant association with a clinical outcome, but no additional information is given and no numerical data are included in any tabular form. These studies were included in the 126 citation results, the full details of which are presented in Supplementary Table 3, to reduce any bias against negative studies. For the subsequent tabular representations of the review findings later, only studies that provide some numerical quantification of their findings regarding lesion location and clinical outcomes are presented to allow for relative comparison to other published literature.

### Supratentorial Versus Infratentorial

Among the studies identified in this review of the literature that met the aforementioned criteria, a supratentorial versus infratentorial anatomical definition was most widely used. The primary outcomes measured in these 47 studies are listed in Table 1. The effect of BM location on OS was measured at a variety of timepoints, with some studies quantifying survival at 6 months, 1 year, 2 years, median survival, or mean survival. Eleven studies did not report a *P* value or confidence interval or looked at clinical end points other than survival (eg, LC or toxicity), but are still included in the table for completeness. The number of reports identifying a survival difference based on a supra- versus infratentorial BM location with at least one time point being statistically significant (α = 0.05) was 10 (21.3%) studies. Of these, nine demonstrated more favorable survival in patients with supratentorial BMs relative to an infratentorial location.

**Table 1.** Studies reviewed that used a binary division of the brain along the tentorium

Name of author	Year of publication	Number of patients	Number of lesions in each arm	Histology of primary	Outcome measure	Significance (*P* value)	Hazard/odds* ratio
Jose Lorenzoni	2004	110	NR	Mixed		.87	Death 0.89 (0.2–3.87)
Maarouf A. Hammoud	1996	100	Supra: 48 Infra: 33 Both: 19	Colon/rectum	Risk ratio for overall survival	Infra: .72 Both: .97	Death: Supra: 1.00 Infra: 1.08 Both: 1.01
Dirk Rades	2012	152	Supra: 116 Infra: 36	Mixed	6-month OS (%): Supra: 78 Infra: 58 1-year OS: Supra: 57 Infra: 46 6-month LC: Supra: 84 Infra: 74 1-year LC: Supra: 62 Infra: 71	6-month OS: .06 1-year OS: .04 6-month LC: .28 1-year LC: .19	
David M. Routman	2018	391	Supra: 245 Infra: 146	Mixed		Infra: .2418	Death: Infra: 1.140 (0.915–1.415)
Cheng Yu	2002	122	Supra: 98 Infra: 24	Melanoma	Median OS (months): Supra: 6.4 Infra: 10.3 6-month OS: Supra: 50.5 (40.3–60.7) Infra: 73.9 (56–91.8) 1-year OS: Supra: 21.5 (13.2–29.8) Infra: 39.1 (18–60.2)	Infra vs. Supra: .025*	
Yukio Saitoh	1999	24	Supra: 16 Infra: 8	Non-small cell lung cancer	Median OS (months): Supra: 7.5 Infra: 5.8	Median OS: .4206	
Yoshimasa Mori	1998	60	Supra: 45 Infra: 15	Melanoma		OS: Infra: .37	
R. J. Andrews	1996	25	Supra: 16 Infra: 9	Non-small cell lung cancer	Mean OS (months): Supra: 14.6 Infra: 10.3	NS	
Marek Wronski	1997	119	Supra: 96 Infra: 23	Renal cell carcinoma	Median OS (months): Supra: 3.3 Infra: 2.4	.12	
Raymond Sawaya	1998	400	Supra: 358 Infra: 42	Mixed		All neurological complication: .32 Regional complications: .030 Systemic complications: .020	All neurological complications: Supra: 1 Infra: 0.51 (0.12–2.22) Regional complications: Supra: 1 Infra: 4.61 (1.32–16.1) Systemic complications: Supra: 1 Infra: 5.28 (1.47–18.9)
Marek Wronski	1999	73	Supra: 47 Infra: 26	Colon/rectum	Mean OS (months): Infra: 6.5 Supra: 13.4 Median OS (months): Infra: 5.1 (3.4–7.7) Supra: 9.1 (7.6–12.4)	.002*	
Frederick Enders	2016	114	Supra: 81 Infra: 16 Both: 16	Non-small cell lung cancer	Median OS (months): Supra: 12.6 (10.3–14.6) Infra: 6.3 (3.5–19.3)	.049	
Narayan Sundaresan	1985	125	Supra: 106 Infra: 19	Mixed	Median OS (months): Supra: 13 Infra: 7		
Jun Hyong Ahn	2012	242	Supra: 204 Infra: 38	Mixed		.18	LMD: Infra: 1 Supra: 0.57 (0.25–1.30)
Rogne SG	2012	316	Supra: 253 Infra: 63	Mixed		>.05	Death: Supra vs. Infra: 1.269 (0.92–1.74)
Eric Ojerholm	2014	91	Supra: 74 Infra: 22	Mixed		LF: Infra vs. Supra: .056 LMD: Infra vs. Supra: .0014	
Eben Alexander III	1995	182	Supra: 345 Infra: 76	Mixed		LC: Infra: .003	RR for LC: 2.51 (1.34–4.69)
P. H. Graham	2010	113	Supra: 77 Infra + Both: 36	Mixed	Median OS (months): Infra or both: 5.7 Supra: 7 Median LC (months): Infra or both: 3.8 Supra: 9.4	OS: .007 LC: <.001	Death: 1.79 (1.2–2.7) RC: 3.16 (1.7–5.8)
Ashley Emery	2017	300	Supra: 609 Cerebellum: 155 Brainstem: 43 Other: 10	Mixed		Brainstem vs. supratentorial: <.001 Cerebellar vs. supratentorial: .46 Brainstem vs. cerebellar: <.001	Death HR: Brainstem vs. supratentorial: 3.52 (1.81–6.85) Cerebellar vs. supratentorial: 1.21 (0.73–1.99) Brainstem vs. cerebellar: 2.92 (1.62–5.26)
Kaisorn Lee Chaichana	2014	708	Infra: 140 Supra: 568	Mixed	Median OS (months): Infra: 8.2 Supra: 9.9 6-month OS (%): Infra: 56.2 Supra: 61.8 1-year OS (%): Infra: 35.3 Supra: 43.1 2-year OS (%): Infra: 21.5 Supra: 27.2 6-month DPFS (%): Infra: 76.3 Supra: 70.6 LR: Infra: 86.9 Supra: 86.4 SR: Infra: 88 Supra: 94.3 1-year DPFS (%): Infra 50.8 Supra: 55.3 LR: Infra: 80.2 Supra: 76	OS: .11 DPFS: .84 LR: .86 SR: .002	
					SR: Infra: 75 Supra: 90.8 2-year DPFS (%): Infra: 42.3 Supra: 41.6 LR: Infra: 73.4 Supra: 68.4 SR: Infra: 75 Supra: 84.9		
Stefan Huttenlocher	2014	214	Supra: 179 Supra + Infra: 35	Mixed	LC: Supra: 63 Supra + Infra: 55	.49	
Dirk Rades	2016	34	Supra: 23 Infra: 11	Breast	1-year OS (%): Supra: 59 Infra: 80 2-year OS (%): Supra: 34 Infra: 39	.32*	
Ivo W. Tremont- Lukats	2003	103	Supra: 78 Infra: 22	Prostate		.66	
Bernardo Cacho Diaz	2018	570	Supra: 282 Infra: 44 Supra + Infra: 158 Carcinomatosis: 88	Mixed	Median OS (months): Supra: 12 (8.9–15.1) Infra: 12 (7.9–16.1) Supra and Infra: 12 (9.7–14.3) Carcinomatosis: 4 (2.3–5.6)	NR	
Dirk Rades	2015	98	Supra: 84 Infra: 14	Lung	6-month freedom from new brain mets: Supra: 68 Infra: 71 1-year freedom from new brain mets: Supra: 46 Infra: 71 2-year freedom from new brain mets: Supra: 25 Infra: 71	.19*	
Todd W. Flannery	2003	72	Supra: 56 Infra: 16	Non-small cell lung cancer	Median OS (months): Supra: 14.3 Infra: 16.4	.871	
Liesa Dziggel	2015	34	Supra: 23 Infra: 11	Non-small cell lung cancer and breast cancer	6-month freedom from new brain mets: Supra: 77 Infra: 82 1-year freedom from new brain mets: Supra: 64 Infra: 82	NS	
Adam A. Garsa	2014	228	Supra: 335 Infra: 66	Non-small cell lung cancer	1-year LC (%): Supra: 77 Infra: 60	HR Infra LR: .13	LR Infra: 1.87 (1.14–3.06)
Marek Wronski	1996	50	Supra: 42 Infra: 8	Renal cell cancer	Median OS (months): Supra: 12 Infra: 3	.63	
Elisa Y. Saito	2006	270	Supra: 140 Infra: 24 Supra + Infra: 47	Mixed	1-year OS (%): Supra: 27 Infra: 18 Both: 25.2	.29	
Marek Wronski	1995	231	Supra: 204 Infra: 27	Non-small cell lung cancer	Mean OS (months): Supra: 24.5 Infra: 12	<.04	
Filippo Pietrantonia	2015	227	Supra: 124 Infra: 103	Colorectal cancer	Median OS (months): Supra: 7 Infra: 4	<.0001	
Jose Marcus Rotta	2018	71	Supra: 59 Supra + Infra: 12	Mixed	Median OS (months): Supra: 19.9 Supra + Infra: 16.1	>.05	
Rasheed Zakaria	2014	76	Supra: 64 Infra: 12	Mixed	Median OS (months): Supra: 9.6 (8.2–11.1) Infra: 4.4 (0–10.1) Median PFS (months): Supra: 17.7 (10.8–24.6) Infra: Not reached	OS: .070 PFS: .872	
Charles A. Sansur	2000	173	Supra: 160 Infra: 33	Mixed	Median OS (months): Supra: 8.7 Infra: 4.4	.03	
Tim J. Kruser	2008	49	Supra: 16 Infra: 25 Supra + Infra: 8	Colorectal cancer	Median OS (months): Supra: 5.7 Infra: 4.5 Supra + Infra: 6.6 6-month OS (%): Supra: 44 Infra: 25 Supra + Infra: 60 1-year OS (%): Supra: 20 Infra: 6 Supra + Infra: 0	Supra vs. Infra vs. Supra + Infra Tumor location: .28* Supra vs. Infra: .14	
Kwan H. Cho	2000	83	Supra: 44 Infra: 6 Supra + Infra: 33	Mixed	Median OS (months): Supra: 7.8 Infra: 6.7 Supra + Infra: 4 1-year OS (%): Supra: 24 Infra: 44 Supra + Infra: 17	.1*	
Carsten Nieder	2016	64	Supra: 34 Supra + Infra: 30	Colorectal cancer	Median OS (months): Supra: 4 Infra or Infra + Supra: 3.6	.86	
Nicolas Penel	2001	124	Supra: 85 Infra: 39	Lung cancer	Median OS (months): Supra: 7.33 +/− 16 days Infra: 8.07 +/− 17 days	.0037	
Dirk Rades	2014	148	Supra: 127 Infra: 21	Lung cancer	6-month OS (%): Supra: 72 Infra: 67 1-year OS (%): Supra: 53 Infra: 51 6-month LC (%): Supra: 93 Infra: 88 1-year LC (%): Supra: 81 Infra: 88	LC: .20 OS: .72 Distant intracerebral control: .39	
					6-month distant intracerebral control: Supra: 77 Infra: 82 1-year distant intracerebral control: Supra: 59 Infra: 72		
Heon Yoo	2009	94	Supra: 75 Infra: 19	Mixed		LR: .403 OS: .638	HR death 1.15 (0.64–2.07) HR LR 1.44 (0.62–3.36)
Katrina S. Firlik	2000	58	Supra: 44 Infra: 14	Breast	OS (values NR)	.53	
Robert A. Badalament	1990	20	Supra: 12 Infra: 6	Renal cell cancer	Median OS (months): Infra: 28 Supra: 12.9	.19	
Hidemitsu Nakagawa	1994	89	Supra: 52 Infra: 5	Lung cancer	Median OS (months): Supra: 16.47 Infra: 7.17	<.05	
A. Fowler	2007	32	Supra: 22 Infra: 10	Colorectal cancer	Mean OS (months): Supra: 9.08 Infra: 6.9 Median OS (months): Supra: 7.7 Infra: 6.37	Supra: .218* Infra: .185*	
Roberta Ruda	2001	33	Supra: 21 Infra: 12	Mixed		.71	HR death 0.93 (0.64–1.35)
Kevin Shiue	2014	320	Supra: NR Infra: NR	Mixed		.431	HR LF: Infra vs. Other 1.891 (1.020–3.507)

Outcome measure in %.

DPFS, disease progression-free survival; HR, hazard ratio; Infra, infratentorial; LC, local control; LF, local failure; LR, local recurrence; LMD: leptomeningeal disease; OS, overall survival; PFS, progression-free survival; RC, regional control; RR, relative risk; Supra, supratentorial; SR, spinal recurrence; NS: nonsignificant; NR, not reported.

*P value given, but with some ambiguity regarding which comparators are being tested.

The only study indicating a survival advantage with infratentorial location was by Penel et al.^6^ and was comprised a heterogeneous population of patients treated with surgical resection, radiation, chemotherapy, or no intervention due to poor prognosis or inoperability. Infratentorial location was associated with a median survival of 8.07 months compared to 7.33 months for supratentorial, a difference of 22 days that was significant on univariable, but not multivariable analysis. This is a relatively small absolute difference in survival and there were a large number of covariates (16) included on multivariable analysis so there may be some susceptibility to lack of statistical power for this analysis. The study by Pietrantonio et al.^7^ found that infratentorial location was an independent predictive of poorer OS in patients with colorectal cancer and led to inclusion of this variable into their proposed nomogram. Taken together, the review of studies analyzing the effect of supra- versus infratentorial location suggests either no impact on OS or an improvement with supratentorial lesions.

An important source of variability both within and across reports is the presence of treatment heterogeneity. Brainstem lesions are unresectable, and when treated with radiosurgery the dose may be limited by concerns over toxicity,^8^ whereas cerebellar BMs are generally amenable to surgery and may be treated highly effectively with either postoperative radiation or with definitive radiosurgery.^9^ Studies that combine brainstem and cerebellar locations into one category (ie, infratentorial) without controlling for local therapy delivered are more difficult to interpret, and it is important to highlight the results of reports where these factors are specifically considered in the analysis. In one such report, Chaichana et al.^10^ attempted to define the prognostic significance of a cerebellar metastasis while carefully evaluating the effect of surgery. The authors found that in the entire cohort of 708 patients, cerebellar location was an independent predictor of decreased survival when compared to supratentorial BMs [RR (95% CI), 1.231 (1.016–1.523)]. However, when the analysis was restricted only to patients who had undergone resection of the cerebellar metastasis, there was no difference in median survival when compared to supratentorial lesions (8.2 vs. 9.9 months; *P* = .11). In another study performed by Trifiletti et al.,^11^ a propensity score matched analysis was performed to compare the survival outcomes in patients with brainstem lesions treated with SRS and a comparator cohort of patients treated with SRS for non-brainstem metastases. After propensity matching of 316 patients, the authors found that brainstem location was associated with a poorer median survival as measured from the date of treatment (4.4 vs. 6.5 months; *P* = .035). Unfortunately, that analysis did not make specific comparisons to cerebellar location, but in a later article by the same authors^12^ and possibly an overlapping patient population, this was addressed. Here, 817 BMs were analyzed with respect to supratentorial, brainstem, or cerebellar location. Again, all patients were treated with SRS and very few (9%) had undergone a prior resection. In this large and relatively homogeneous population, OS with brainstem location was significantly worse when compared to supratentorial [HR for death (95% CI), 3.52 (1.81–6.85)] and cerebellar [HR 2.92 (1.62–5.26)] location. Yet, there was no significant difference in the comparison of cerebellar and supratentorial location [HR 1.21 (0.73–1.99)]. In contrast to these findings, Hasegawa et al.^13^ reviewed their experience with a relatively a homogeneous population of patients treated with SRS alone where 80% of the cohort had only a single BM (Table 2). Here, the authors specifically analyzed the effect of brainstem and cerebellar location on OS and found no evidence that either site influenced outcomes.

**Table 2.** Studies reviewed with an anatomical definition other than supra- vs. infratentorial or by lobes of the brain

Name of author	Year of publication	Number of patients	Number of lesions in each arm	Histology of primary	Outcome measure	Significance (*P* value)	Hazard/odds ratio
B. H. Kye	2012	39	Cerebrum: 23 Cerebellum: 7 Both: 9	Colorectal cancer	Median OS (months): Cerebrum: 5 +/− 1.1 Cerebellum: 6.5 +/− 4.6 Both: 4.3 +/− 1.9	.254	
Hitoshi Ikushima	2000	33	Eloquent: 3 Non-eloquent: 30	Renal cell cancer	Median OS (months): Eloquent: 21.2 Non-eloquent: 18	.05	
T. Shuto	2003	25	Midbrain: 7 Pons: 15 Medulla: 1	Mixed	6-month LC (%): Midbrain: 90 Pons: 74 Medulla: 100	NR	
Bradley M. Swinson	2008	619	Eloquent: 273 Non-eloquent: 346	Mixed		OS: .687 RC: .026	HR death: 0.962 (0.812–1.14) HR RC: 1.672 (1.048–2.67)
S. Meier	2004	100	Cerebrum: 78 Cerebrum + Other: 18	Melanoma	Median OS (months): Cerebrum: 5.4 Cerebrum and other: 2.6 6-month OS (%) Cerebrum: 39 Cerebrum and other: 27 1-year OS (%): Cerebrum: 15 Cerebrum and other: 13 2-year OS (%): Cerebrum: 6 Cerebrum and other: 0	0.11	
Gerd Becker	2002	41	Midline: 3 Other: 38	Mixed	Median OS (months): Midline: 7 Other: 7 1-year OS (%): Midline: 33 Other: 31 2-year OS (%): Midline: 0 Other: 18 Median LC (months): Midline: 3 Other: 15 1-year LC (%): Midline: 33 Other: 55 2-year LC (%): Midline: — Other: 48	OS: .713 LC: .0837	
Fred Hsu	2015	212	Eloquent: 188 Non-eloquent: 24	Mixed	Median OS (months): Eloquent: 16.4 Non-eloquent: 10.8	.16	
Robert E. Elliott	2010	98	Eloquent: 111 Non-eloquent: 96	Mixed		.027	OR: Neurological complication: Eloquent: 6.59
Caroline Gaudy- Marqueste	2006	106	Cortical: 33 Subcortical: 159 Cerebellum: 16 Brain stem, nuclei, posterior fossa: 13	Melanoma	Median OS (months): Cortical: 5.52 (1.29–9.75) Subcortical: 6.08 (4.66–7.50) Cerebellum: 3.44 (1.20–5.68) Brain stem, nuclei, posterior fossa: 2.18 (1.87–2.49)	.0003	
Toshinori Hasegawa	2003	172	Solitary lesions Brainstem: 1 Cerebellum: 22 Cerebrum: 113 Multiple lesions and Other: 36	Mixed		OS Brainstem: .1346 Cerebellar: .2205	
Toshinori Hasegawa	2003	39	Lobar: 21 Non-lobar: 18	Upper GI cancer	Median OS (months): Lobar: 5 Non-lobar: 8		HR death: Non-lobar: 0.74 (0.36–1.50)
Satoshi Maesawa	2000	15	Brainstem: 4 Non-brainstem: 26	Mixed	1-year OS (%): Brainstem: 25% Non-brainstem: 82% 2-year OS (%) Brainstem: 0 Non-brainstem: 73%	.004	
Takeaki Ishihara	2016	53	Eloquent: 18 Non-eloquent: 58	Lung cancer	1-year LC (%): Eloquent: 78.7 Non-eloquent: 85.1	.808	
Anthony L. Asher	2013	47	Frontal: 5 Parietal: 5 Temporal: 2 Occipital: 2 Cerebellum: 3 Pons: 3	Mixed		Eloquent: .52	HR death: Eloquent: 1.342 (0.549–3.281)

GI, Gastrointestinal; HR, hazard ratio; LC, local control; NR, not reported; OR, odds ratio; OS, overall survival; RC: regional control.

Other than differences in survival, several retrospective investigations have identified a relationship between infratentorial location and other outcomes, including local or regional control^10,14–24^ and LMD.^15,25,26^ Of the 12 studies, we identified that measured effects on intracranial disease control only two^16,17^ found a significant difference in LC, both in favor of supratentorial location. In regard to LMD, two of three studies we identified that specifically investigated this measure found an increased risk with infratentorial BMs.^15,26^

In summary, the comparison of supratentorial versus infratentorial location has been the most well studied, but there are conflicting results from a diverse array of publications. Importantly, infratentorial location includes both the brainstem and cerebellum, which are diametrically opposed in terms of resectability and this may impart some effect on patient outcomes. It is difficult to interpret reports that include patients treated with a variety of interventions and yet do not separately analyze brainstem location, resected cerebellar lesions, and unresected cerebellar lesions. Our literature review identified several studies that attempted to account for these important factors and there is a suggestion that brainstem location and unresected cerebellar lesions negatively affect survival, but we note that it is difficult to draw conclusions from a highly selected set of publications. Further, infratentorial location might be associated with a reduced local and distant in-brain disease control and may increase the risk of LMD, but the literature is particularly limited in these outcome measures.

### Lobar Classification

Another commonly used anatomical categorization for the data was a lobar classification. The primary outcomes of these seven studies are listed in Table 3. The effect of BM location on survival was measured as median survival, progression-free survival, or 2-year OS. One study specifically investigated complication rates based on BM location^27^ and is also included in Table 3. There were five studies with no statistically significant difference in survival and we identified only one study^28^ where a significant survival difference was found. In this study, 89 patients who exclusively had a primary diagnosis of melanoma were compared based on a frontal (*N* = 61) versus non-frontal lobe (*N* = 28) BMs and results indicated a more favorable median survival in patients with non-frontal lobe lesions (4.9 vs. 10 months, *P* = .01).

**Table 3.** Studies reviewed that used a lobar segregation of the brain

Name of author	Year of publication	Number of patients	Number of lesions in each arm	Histology of primary	Outcome measure	Significance (*P* value)/hazard ratio
W. A. Hall	2000	740	Frontal: 137 Parietal: 43 Temporal: 83 Occipital: 41 Cerebellum: 72	Mixed	2-year OS (%): Frontal: 10 Parietal: 26 Temporal: 2 Occipital: 10 Cerebellum: 7	2-year OS: Frontal: .448 Parietal: .706 Temporal: .115 Occipital: .788 Cerebellum: .761
Stephane Culine	1998	68	Frontal: 24 Parietal: 34 Occipital: 15 Temporal: 18 Cerebellum: 13	Renal cell cancer	Median OS (month): Frontal: 5 Parietal: 7 Occipital: 7 Temporal: 6 Cerebellum: 7	Frontal: .5 Parietal: .2 Occipital: .8 Temporal: .06 Cerebellum: .4
Jan Zakrzewski	2011	89	Frontal: 61 Non-frontal: 28	Melanoma	Median OS (month): Frontal: 4.9 Non-frontal: 10	Median OS: .01
Stefan Huttenlocher	2014	69	Frontal: 23 Temporal: 17 Other: 29	Melanoma	PFS (%): Frontal: 59 Temporal: 64 Other: 50	PFS: .36
Young Soo Kim	1997	77	Frontal: 28 Parietal: 38 Occipital: 7 Temporal: 16 Cerebellum: 19	Non-small cell lung cancer		OS: .12
Yoshimasa Mori	1998	35	Frontal: 13 Parietal: 12 Occipital: 7 Temporal: 10 Cerebellum: 2	Renal cell cancer		OS: Lobar lesion: .45
Brian J. Williams	2009	273	Frontal: 125 Parietal: 46 Occipital: 31 Temporal: 30 Cerebellum: 39 Brainstem: 17 Other: 28	Mixed	OC: Eloquent: <0.001 Brainstem: 0.006 Motor, sensory, visual, speech: 0.01 NC: Eloquent: < 0.001 Brainstem: 0.02 Motor, sensory, visual, speech: 0.001	HR (NC): Eloquent: 2.6 (1.7–3.8) Brainstem: 2.2 (1.2–4.3) Motor, sensory, visual, speech: 2 (1.3–3.1) HR (OC): Eloquent: 2.4 (1.7–3.4) Brainstem: 2.2 (1.2–4) Motor, sensory, visual, speech: 1.6 (1.1–2.2)

HR, hazard ratio; NC: Neurological Complications; OC, other complications; OS, overall survival; PFS, progression-free survival.

**Figure 2.**
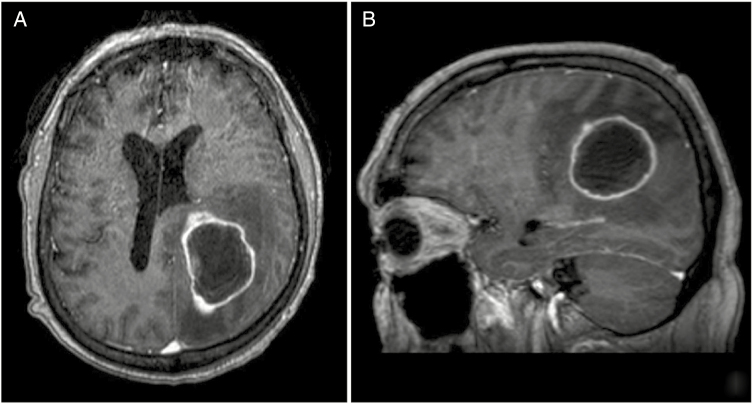
A single, cystic BM in a patient with transitional cell carcinoma of the urinary bladder measuring up to 4.7 cm in the left parietal white matter and involving multiple lobes in the brain, including the left parietal and occipital cortices (**A**) and (**B**). The patient presented with right upper extremity weakness, suggesting impairment of the left posterior frontal cortex. How a BM with anatomical involvement of two lobes (parietal and occipital) and functional impairment of a third (frontal) is categorized across studies is variable and may contribute to disagreement in the literature on how BM location affects clinical outcomes. BM, brain metastasis.

There are several limitations to studies using a lobar definition to quantify the effect of BM location on clinical outcomes. First, more than one lobe is often involved with a single lesion and a BM might be characterized by the lobe thought to be most affected based on radiographic appearance or the presence of symptoms, by what is considered to be the center or origin of the tumor, or some other subjective means of classification (Fig. 2). Along the same lines, a single patient may have multiple BMs involving more than one lobe and it is unclear how that patient should be categorized in a regression analysis. Further, the precise boundaries of how each lobe is anatomically defined may be subjective, with some variation expected between investigators. Finally, five of the seven studies outlined in Table 3 were performed with less than 100 patients (median number of patients = 77, range 35–740) which places limits on the statistical power to detect a difference in survival with a greater number of anatomic subunits. Therefore, the inherent ambiguity of a lobar anatomical definition both as it pertains to normal anatomy, categorization of a single lesion, and categorization of patients with multiple BMs across more than one lobe make this approach unpromising. In the reported literature, relatively small sample sizes further limit our ability to draw conclusions about how the presence of a metastasis in different lobes of the brain might affect clinical outcomes.

### Other Methods of Classification

Amidst the remaining studies, eloquent versus non-eloquent (*n* = 5) was a frequently used anatomical definition. The remaining studies (*n* = 9) with other anatomical definitions (eg, midbrain classifications) were summarized together in Table 2. The effect of BM location on OS or LC was measured at a variety of timepoints, with some studies quantifying survival at 6 months, 1 year, 2 years, or median. There was only one study^29^ with survival difference based on an eloquent versus non-eloquent BM location that was statistically significant (α = 0.05). Ikushima et al. demonstrated a slightly more favorable survival in patients with eloquent BMs (*N* = 3) relative to non-eloquent (*N* = 30) location (median survival 21.2 months vs. 18 months, *P* = .05). With an overall small sample size and only three patients with a BM in an eloquent location, the difference of approximately 3 months in median survival is of questionable significance. Eloquent location of a BM has an inherent effect on treatment decisions and studies evaluating clinical outcomes with these lesions must be considered with the same caveats discussed earlier for infratentorial (brainstem vs. cerebellum) metastases. Namely, neurologic complications are more likely either at presentation or subsequent to local therapy for BMs in an eloquent location, and this has been demonstrated empirically even in small lesions.^30^

As discussed in detail earlier, comparisons of supra- versus infratentorial have several limitations and some studies in Table 2 analyzed BM location in a way that addresses some of these. In particular, some authors analyzed the potentially meaningful division of the infratentorial structures into brainstem and cerebellum. In one study, median survival was highest in cortical and subcortical locations, lower in the cerebellum, and lowest in the brainstem, although the anatomical nomenclature of how the posterior fossa was anatomically segregated is not entirely clear.^31^ Another report found brainstem location to have a dramatically worse 1-year and 2-year OS compared to non-brainstem location, but this was in a very small sample size.^32^ Aside from these two and as discussed earlier, Hasegawa et al.^13^ specifically analyzed the brainstem and cerebellum separately and found no evidence of an effect on survival.

**Figure 3.**
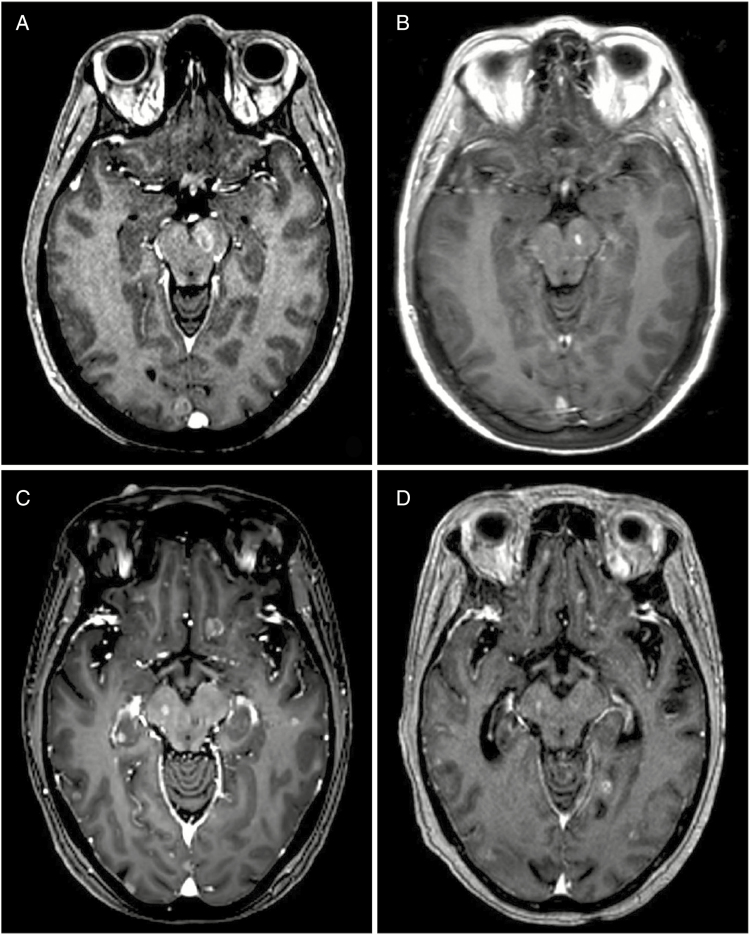
A patient with EGFR-mutant NSCLC presenting with a BM in the left cerebral peduncle and two other supratentorial BMs (**A**). All lesions were treated with SRS and remained controlled at 6 months (**B**). A similar patient with EGFR-mutant NSCLC with innumerable (>50) BMs throughout the supra- and infratentorium, including one lesion in the right cerebral peduncle (**C**). The patient was treated with WBRT and most lesions were well controlled, including the midbrain focus, but some lesions appeared to progress at seven months follow-up (**D**). Whether brainstem involvement itself portends a poorer prognosis and/or predicts for more diffuse intracranial disease could not be resolved in this review of the literature. BM, brain metastasis; EGFR, epidermal growth factor receptor; NSCLC, non-small cell lung cancer; SRS, stereotactic radiosurgery; WBRT, whole-brain radiotherapy.

One explanation for the variability in findings even among studies that specifically analyzed brainstem location might relate to the presence of other lesions outside the brainstem. For example, it is unclear if the presence of a single metastasis within the brainstem is still more favorable than a patient with diffuse BMs where the brainstem is involved. It is well documented that the brainstem is at a low risk of harboring disease relative to the cerebellum and most cortical structures.^33^ Therefore, a population of patients with brainstem lesions might represent a poorer group because of a higher overall intracranial burden of disease, which makes it difficult to interpret studies that do not control for the number or volume of BMs in addition to lesion location (Fig. 3).

In summary, a number of investigators delineated anatomical boundaries based on clinical relevance in an attempt to identify BMs that may present a threat to critical neurologic function. There is little evidence that eloquent location portends a worse survival, but there is an inherent relationship to neurologic function, which may be used to guide management decisions. Considering the only other significant findings were with some variation of supra- versus infratentorial location (eg, cortical vs. brainstem vs. cerebellum), we found no strong evidence to support any other anatomical division(s) of the brain with respect to any clinical outcome.

## Discussion

BMs are a neurological complication of cancer that results in significant morbidity and mortality. Although most patients have a limited life expectancy of several months, prognosis of patients with BMs is variable and depends on numerous patient- and cancer-specific factors.^34,35^ A thorough understanding of these variables is not only important to inform patients, but also guide treatment decisions.

A number of prognostic scoring systems for BM patients have been proposed using a variety of patient populations and has recently been reviewed elsewhere.^2^ Recursive partitioning analysis (RPA), one of the earliest and most widely used systems that was developed by Radiation Therapy Oncology Group established four prognostic variables: age (≥65 vs. <65), Karnofsky Performance Status (KPS; ≥70 vs. <70), control of primary tumor, and the presence of extracranial metastases.^36^ The Score Index for Radiosurgery was a similar proposal to the RPA but incorporated the volume of the largest BM (<5 vs. 5–13 vs. >13 cc) given the importance of this variable to SRS planning.^37^ Of the early scoring systems, the Basic Score for Brain Metastases was perhaps the simplest with only three binary variables (KPS: 50–70 vs. 80–100; control of primary tumor: Yes vs. No; extracranial metastases: Yes vs. No).^38^ Another prognostic index, Graded Prognostic Assessment (GPA), was intended to address the limitations of previous nomograms and has since supplanted these in most clinical practices with refinements made over time. Analysis of age, sex, KPS, histologic characteristics, interval from initial diagnosis to time of presentation with BMs, number of BMs, and patients with brain and bone-only metastases were used to derive the GPA. On multivariable analysis, only age (*>*60 vs. 50–59 vs. *<*50), KPS (*<*70 vs. 70–80 vs. 90–100), presence of extracranial metastases (Yes vs. No), and number of BMs (1 vs. 2–3 vs. *>*3) were significant and, therefore, were included in the scoring system.^39^ The selection of factors for the original GPA was performed to remove the subjectivity in assessing control of systemic disease and include quantifiable measures of BM burden (ie, number of metastases) without relying on treatment factors (ie, volume of the largest lesion at the time of SRS).^39^ The GPA has been further developed over time with major modifications including the generation of disease-specific scores (DS-GPA) which addressed the difference among primary malignancies^40,41^ and the addition of important molecular and histologic data, such as receptor status for breast cancer^3^ and genetic variants in NSCLC.^4^ There is also evidence that certain primary histologies have characteristic spatial patterns of BMs and it would be interesting to understand how location might have a differential impact on outcomes based on the origin of the primary tumor.^42–44^ However, our review identified too few studies evaluating location-specific clinical outcomes based on primary tumor histology to make meaningful comparisons across different histologies. For example, the most common exclusively studied histology was NSCLC (14 studies, 11.1% of the total included) and only five of these reported OS based on BM location.

As discussed earlier, there are many challenges in determining how the location of a BM might affect patient outcomes in institutional series, including variability in how anatomic regions are defined, controlling for differences in local interventions, and the statistical demands of including numerous brain regions in multivariable analyses. Additional limits may influence the development of large-scale prognostic scores, which often rely on data drawn from multicenter databases or records gathered from cooperative group studies where granular anatomic neuroimaging information might not be readily available. Despite the absence of BM location from commonly used prognostic scoring systems, it is used frequently in clinical practice and location generally affects decisions regarding local therapy. In terms of the published literature, some authors have argued strongly that posterior fossa lesions portent a worse prognosis, with risk of brainstem compromise, hydrocephalus, and cerebellar herniation leading to neurologic decrement, suggesting that the intervention of choice for these lesions might be surgery with or without radiation^9,45,46^ or SRS.^47^ For these reasons, consensus guidelines from the American College of Radiology considers location as one of three key components to consider, in addition to the number and size of lesion(s), in the pretreatment evaluation of a patients with BMs.^48^

This review is limited by several factors. First, there are caveats with the interpretation of any individual retrospective study and these are inherent to our summary of this literature. We are particularly limited by the correlation between BM location and surgical intervention that is present across our literature review. The brainstem presents the clearest example as it can be assumed that a BM in this location is unresectable and if untreated will lead to significant morbidity and ultimately mortality. But can aggressive nonsurgical treatment provide sufficient LC while avoiding prohibitive toxicities to offset this poor prognosis? There were conflicting findings across the literature regarding the brainstem, but some evidence suggests that even with aggressive treatment, survival is still decreased with this unfavorable location. For other areas of the brain, the relationship between BM location and prognosis is even more obscure because resectability and the implementation of other forms of therapy are more variable. We were unable to disentangle this relationship between location and treatment and recommend that future investigators use cohort studies matching patients for these variables. Although additional studies specifically comparing brainstem location with the cerebellum or supratentorial lesions are needed, matched cohort studies focused on the motor cortex, thalamus, language areas, or other regions of the brain with control of eloquent function would be particularly informative.

## Conclusions

In this review of the literature, we identified numerous studies evaluating prognostic variables in patients with BMs with many of these specifically testing the relevance of the anatomical location of the lesion. The means of subdividing the brain was variable as were the effects of BM location on clinical outcomes, including survival, disease control, and toxicities. Although there was no clear consensus in the literature, the majority of studies either found no evidence of an effect on survival or suggested that lesions in the brainstem and cerebellum portend a worse prognosis. The primary literature and well-validated prognostic scores are not entirely congruent with basic principles of neuro-oncology, which dictate that the anatomical location of a BM influences treatment decisions and should be considered when assessing patient prognosis. Still, without high-level evidence it is unclear what affect BM location has on key clinical outcomes and large-scale prognostic indices should attempt clarify this ambiguity.

## Funding

There was no source of funding for this literature review.

## Supplementary Material

vdz017_suppl_Supplementary_Table_1

vdz017_suppl_Supplementary_Table_2

vdz017_suppl_Supplementary_Table_3

## Notes

**Authorship statement:** Review of the literature (P.K. and T.K.Y.). Data summaries of the literature review (A.I.). Study concept and manuscript preparation (all authors). All authors have approved the final version of this manuscript.
