# Supplementary material for: The effect of brain metastasis location on clinical outcomes: A review of the literature
Source: Neurooncol Adv. 2019 Sep 13;1(1):vdz017. doi: 10.1093/noajnl/vdz017 (PMC7212918; doi:10.1093/noajnl/vdz017)
Supplement: vdz017_suppl_Supplementary_Table_1 [file vdz017_suppl_supplementary_table_1.docx]

|  | **Concept: Brain metastasis** | **Concept: Location** | **Concept: Patient outcomes** |
| --- | --- | --- | --- |
| Thesaurus Terms / Subheadings | Brain Neoplasms/secondary [Majr] | N/A | Prognosis  Treatment Outcome  Disease-Free Survival  Progression-Free Survival  Response Evaluation Criteria in Solid Tumors  Radiation Injuries  Meningeal Carcinomatosis  Mortality  Survival Rate  /mortality |
| Text words | brainstem  brain stem  thalamus  cerebral  cerebellum  frontal lobe  parietal lobe  occipital lobe  temporal lobe  supratentorial  infratentorial  motor cortex  AND  secondary  metastas*  metastat* | location  sublocation  site  area  region  structure  substructure  distribution  eloquen*  non eloquen*  noneloquen* | prognosis  outcome  survival  response evaluation  radiation injur*  radiation necrosis  radionecrosis  mortality  leptomeningeal carcinomatosis  meningeal carcinomatosis |

Supplemental Table 1: PubMed/MedLine search terms organized by three broad concepts related to the overall search strategy.

*Asterix represents a “wildcard” to capture multiple related entries (e.g. metastas* = metastasis or metastases) from one search string.
