# Supplementary material for: The effect of brain metastasis location on clinical outcomes: A review of the literature
Source: Neurooncol Adv. 2019 Sep 13;1(1):vdz017. doi: 10.1093/noajnl/vdz017 (PMC7212918; doi:10.1093/noajnl/vdz017)
Supplement: vdz017_suppl_Supplementary_Table_2 [file vdz017_suppl_supplementary_table_2.docx]

Supplemental Table 2: Summary of database queries.

| Database | Search term | Articles Identified | Articles Reviewed |
| --- | --- | --- | --- |
| PubMed/MEDLINE | See Supplemental Table 1 | 665 | 665 |
| PubMed/Non-MEDLINE | See Supplemental Table 1 | 199 | 199 |
| PubMed/MEDLINE using the “Best Match” query option | “brain metastasis location” | 953 | 953 |
| Google Scholar | “brain metastasis location” | 350,000 | 200 |
| Google Scholar | “brain metastases prognosis supratentorial OR infratentorial OR thalamus OR brainstem OR lobe -glioblastoma -astrocytoma -glioma” | 34,800 | 200 |
