# Supplementary material for: The effect of brain metastasis location on clinical outcomes: A review of the literature
Source: Neurooncol Adv. 2019 Sep 13;1(1):vdz017. doi: 10.1093/noajnl/vdz017 (PMC7212918; doi:10.1093/noajnl/vdz017)
Supplement: vdz017_suppl_Supplementary_Table_3 [file vdz017_suppl_supplementary_table_3.docx]

Supplemental Table 3: Full list of studies satisfying inclusion and exclusion criteria for further review.

| First Author | Year of Publication | Analysis Interval | Type of Study | Number of Patients | Tumor Localization | Institutions | Histology |
| --- | --- | --- | --- | --- | --- | --- | --- |
| A Fowler | 2007 | 1999- NR | Retrospective | 32 | NR | Multi | Colon/Rectum |
| Adam A Garsa | 2014 | 1998-2011 | Retrospective | 228 | MRI | Single | NSCLC |
| Aida Ramos Antuna | 2018 | 2002-2015 | Retrospective | 71 | Multimodal | Single | NSCLC |
| Ashley Emery | 2017 | 1995-2014 | Retrospective | 300 | Multimodal | Single | Various |
| Banu Atalar | 2013 | 1998-2011 | Retrospective | 165 | Multimodal | Single | Various |
| Bernardo Cacho Diaz | 2018 | 2010-2017 | Retrospective | 570 | MRI | Single | Various |
| Bobby E. Harrison | 2003 | 1990-1997 | Retrospective | 65 | Multimodal | Single | Melanoma |
| Carsten Nieder | 2016 | NR | Retrospective | 64 | Multimodal | Multi | Colon/Rectum |
| Charles A. Sansur | 2000 | 1992-1997 | Retrospective | 173 | MRI | Single | Various |
| Cheng Yu | 2002 | 1994-1999 | Retrospective | 122 | MRI | Single | Melanoma |
| David M Routman | 2018 | 1994-2015 | Retrospective | 391 | MRI | Single | Various |
| Dirk Rades | 2012 | 1999-2009 | Retrospective | 152 | MRI | Multi | Various |
| Dirk Rades | 2016 | NR | Retrospective | 34 | NR | Single | Breast |
| Dirk Rades | 2015 | 2000-2014 | Retrospective | 98 | Multimodal | Multi | NSCLC/SCLC |
| Dirk rades | 2014 | NR | Retrospective | 148 | NR | Single | NSCLC/SCLC |
| Eben Alexander III | 1995 | 1986-1993 | Retrospective | 182 | CT | Single | Various |
| Eben Alexander III | 1996 | 1986-1995 | Retrospective | 381 | Multimodal | Multi | Various |
| Eduardo Weltman | 2000 | 1993-1997 | Retrospective | 65 | Multimodal | Single | Various |
| Elisa Y. Saito | 2006 | 1996-2000 | Retrospective | 270 | Multimodal | Single | Various |
| Emmanouil Fokas | 2011 | 1996-2007 | Retrospective | 78 | Multimodal | NR | Colon/Rectum |
| Eric Ojerholm | 2014 | 2007-2013 | Retrospective | 91 | MRI | Single | Various |
| Evert M Noordijk | 1994 | 1985-1990 | Retrospective | 63 | CT | Multi | Various |
| Filippo Pietrantonia | 2015 | 2000-2013 | Retrospective | 227 | Multimodal | Multi | Colon/Rectum |
| Frederick Enders | 2016 | 2002-2013 | Retrospective | 114 | MRI | Single | NSCLC |
| Friedrich Weber | 1996 | 1983-1991 | Retrospective | 133 | Multimodal | Single | Various |
| Gabriela Simonova | 2000 | 1992-1998 | Retrospective | 237 | Multimodal | Single | Various |
| Heon Yoo | 2009 | 2001-2007 | Retrospective | 94 | Multimodal | Multi | Various |
| Hidemitsu Nakagawa | 1994 | 1978-1990 | Retrospective | 89 | CT | Single | NSCLC/SCLC |
| Hun Jin Kim | 2013 | 1987-2009 | Retrospective | 38 | Multimodal | Single | Colon/Rectum |
| Ivo W Tremont-Lukats | 2003 | 1944-1988 | Retrospective | 103 | Multimodal | Single | Prostate |
| Jose Lorenzoni | 2004 | 1999-2003 | Retrospective | 110 | MRI | Single | Various |
| Jose Marcus Rotta | 2018 | 2011-2014 | Retrospective | 71 | NR | Single | Various |
| Jun Hyong Ahn | 2012 | 2001-2009 | Retrospective | 242 | MRI | Single | Various |
| Kaisorn Lee Chaichana | 2014 | 1997-2011 | Retrospective | 708 | MRI | Single | Various |
| Katrina S. Firlik | 2000 | 1990-1997 | Retrospective | 58 | Multimodal | Single | Breast |
| Kevin Shiue | 2014 | 2007-2011 | Retrospective | 320 | MRI | Single | Various |
| Kwan H. Cho | 2000 | 1991-1999 | Retrospective | 83 | CT | Single | Various |
| Liesa Dziggel | 2015 | NR | Retrospective | 34 | Multimodal | Single | Breast/NSCLC |
| Maarouf A Hammoud | 1996 | 1980-1994 | Retrospective | 100 | Multimodal | Single | Colon/Rectum |
| Marek Wronski | 1997 | 1976-1993 | Retrospective | 119 | CT | Single | RCC |
| Marek Wronski | 1999 | 1974-1993 | Retrospective | 73 | Multimodal | Single | Colon/Rectum |
| Marek Wronski | 1996 | 1974-1993 | Retrospective | 50 | Multimodal | Single | RCC |
| Michael A Vogelbaum | 2006 | 1997-2003 | Retrospective | 202 | MRI | Single | Various |
| Narayan Sundaresan | 1985 | 1978-1982 | Retrospective | 125 | CT | Single | Various |
| Nicolas Penel | 2001 | 1985-1993 | Retrospective | 124 | CT | Single | NSCLC/SCLC |
| P.H. Graham | 2010 | 1996-2006 | Post-hoc | 113 | Multimodal | Single | Various |
| Paolo Macchiarini | 1991 | 1975-1988 | Retrospective | 37 | CT | Single | NSCLC |
| Pierre Bonnette | 2001 | 1985-1998 | Retrospective | 103 | Multimodal | Multi | NSCLC/SCLC |
| R J Andrews | 1996 | 1986-1993 | Retrospective | 25 | CT | Single | NSCLC |
| Rasheed Zakaria | 2014 | 2007-2012 | Retrospective | 76 | MRI | Single | Various |
| Raymond Sawaya | 1998 | 1992-1994 | Retrospective | 400 | Surgical | Single | Various |
| Robert A Badalament | 1990 | 1976-1986 | Retrospective | 20 | Multimodal | Single | RCC |
| Roberta Ruda | 2001 | 1987-1996 | Post-hoc | 33 | CT | Single | Unknown primary |
| SG Rogne | 2014 | 2003-2011 | Retrospective | 140 | MRI | Single | Various |
| Shinkichi Takamori | 2018 | 2005-2016 | Retrospective | 34 | CT | Single | NSCLC |
| Shoji Yomo | 2012 | 2009-2011 | Post-hoc | 29 | Multimodal | Single | Various |
| Stefen Huttenlocher | 2014 | NR | Retrospective | 214 | Multimodal | Single | Various |
| Tim J. Kruser | 2008 | 1994-2005 | Retrospective | 49 | Multimodal | Single | Colon/Rectum |
| Todd W Flannery | 2003 | 1992-1999 | Retrospective | 72 | MRI | Multi | NSCLC |
| Xiao-Dong Gu | 2015 | 2001-2011 | Retrospective | 93 | NR | Single | Colon/Rectum |
| Yoshimasa Mori | 1998 | 1988-1996 | Retrospective | 60 | Multimodal | Single | Melanoma |
| Yoshiyasu Iwai | 2007 | 1998-2005 | Retrospective | 21 | Surgical | Single | Various |
| Yukio Saitoh | 1999 | 1977-1993 | Retrospective | 24 | Surgical | Single | NSCLC |
| Adela Wu | 2017 | 1998-2011 | Retrospective | 565 | NR | Single | Various |
| Andrea L. Cheville | 2017 | NR | Retrospective | 66 | NR | Single | NSCLC/SCLC |
| Ann A Shi | 2006 | 1996-2003 | Retrospective | 181 | Multimodal | Single | NSCLC/SCLC |
| Audrey Keller | 2017 | 2008-2015 | Retrospective | 181 | MRI | Multi | Various |
| Ching-yeh Hsiung | 1998 | 1987-1994 | Retrospective | 159 | CT | Multi | NSCLC/SCLC |
| Christopher J Carrubba | 2009 | 2002-2007 | Retrospective | 37 | NR | Multi | Melanoma |
| Douglas Kondziolka | 2005 | 1988-2000 | Retrospective | 44 | MRI | Single | Various |
| Fang-Chun Ko | 1999 | 1970-1996 | Retrospective | 53 | Multimodal | Single | Colon/Rectum |
| Hiromasa Kobayashi | 2017 | 1985-2014 | Retrospective | 232 | Surgical | Single | Various |
| Jan Zakrzewski | 2011 | 2002-2008 | Retrospective | 89 | Multimodal | Single | Melanoma |
| Jason P. Sheehan | 2002 | 1987-2001 | Retrospective | 273 | Multimodal | Single | NSCLC |
| John P Mongan | 2009 | 1984-2006 | Retrospective | 39 | Multimodal | Single | Colon/Rectum |
| Josa M. Frischer | 2016 | NR | Retrospective | 61 | Multimodal | Single | Various |
| Kengo Ogura | 2012 | 2008-2011 | Retrospective | 39 | MRI | Single | Various |
| L. Pellettieri | 1987 | 1963-1982 | Retrospective | 134 | NR | Single | Various |
| M.C. Korinth | 2002 | 1989-1996 | Retrospective | 187 | NR | NR | Various |
| Moshe H. Maor | 2000 | NR | Retrospective | 84 | NR | Single | Various |
| Patrick S Swift | 1993 | 1979-1983 | Retrospective | 779 | CT | Single | Various |
| Peter Lindvall | 2009 | 1993-2004 | Retrospective | 59 | NR | Single | Various |
| Samuel M Shin | 2015 | 2006-2013 | Retrospective | 56 | Multimodal | Single | Various |
| Stefan Huttenlocher | 2014 | NR | Retrospective | 69 | NR | Single | Melanoma |
| Stephane Culine | 1998 | 1975-1993 | Retrospective | 68 | Multimodal | Single | RCC |
| Yoshimasa Mori | 1998 | 1988-1996 | Retrospective | 35 | Multimodal | Single | RCC |
| Young Soo Kim | 1997 | 1988-1995 | Retrospective | 77 | Multimodal | Single | NSCLC |
| B. H. Kye | 2012 | 1997-2006 | Retrospective | 39 | Surgical | Multi | Colon/Rectum |
| Lalit kumar | 2003 | 1991-2001 | Retrospective | 18 | Multimodal | Single | GYN |
| Abe E Sahmoun | 2005 | 1989-2002 | Retrospective | 230 | Multimodal | Single | SCLC |
| Ajay Niranjan | 2010 | 1988-2007 | Retrospective | 29 | MRI | Single | Various |
| Anthony L. Asher | 2013 | 2005-2008 | Post-hoc | 47 | Surgical | Multi | Various |
| Bradley M Swinson | 2008 | 1989-2006 | Retrospective | 619 | MRI | Single | Various |
| Brian J. Williams | 2009 | 1993-2004 | Retrospective | 273 | Multimodal | Single | Various |
| Caroline Gaudy-Marqueste | 2006 | 1997-2003 | Retrospective | 106 | CT | Single | Melanoma |
| Daniel M trifilleti | 2016 | 1992-2014 | Retrospective | 316 | Multimodal | Single | Various |
| Fred Hsu | 2015 | 2000-2012 | Retrospective | 212 | Multimodal | Single | Various |
| Gerd Becker | 2002 | NR | Retrospective | 41 | Multimodal | Single | Various |
| Hitoshi Ikushima | 2000 | 1983-1998 | Retrospective | 33 | Multimodal | Single | RCC |
| Koji Takano | 2015 | 2006-2014 | Retrospective | 200 | Multimodal | Single | NSCLC and SCLC |
| Marek Wronski | 1995 | 1976-1991 | Retrospective | 231 | Multimodal | Single | NSCLC |
| Michael L. DiLuna | 2007 | 1998-2004 | Retrospective | 334 | Surgical | Multi | Various |
| Paul J Kelly | 2011 | 2001-2009 | Retrospective | 24 | MRI | Single | Various |
| Robert E. Elliott | 2010 | 2001-2009 | Retrospective | 98 | MRI | Single | Various |
| S Meier | 2004 | 1966-2002 | Retrospective | 100 | Multimodal | Multi | Melanoma |
| T Shuto | 2003 | 1992-2001 | Retrospective | 25 | Multimodal | Single | Various |
| Takeaki Ishihara | 2016 | 2007-2013 | Retrospective | 53 | Multimodal | Single | NSCLC and SCLC |
| Toshinori Hasegawa | 2003 | 1987-2001 | Retrospective | 172 | Multimodal | Single | Various |
| Toshinori Hasegawa | 2003 | NR | Retrospective | 39 | MRI | Single | GI |
| W Boogerd | 1993 | 1980-1990 | Retrospective | 137 | CT | Single | Breast |
| William D Tobler | 1994 |  | Retrospective | 17 | Multimodal | Single | Various |
| John T Lucas | 2015 | 1999-2012 | Retrospective | 293 | Multimodal | Single | Various |
| Rogne SG | 2012 | 2005-2009 | Retrospective | 316 | MRI | Single | Various |
| Satoshi Maesawa | 2000 | 1988-1998 | Retrospective | 15 | Multimodal | Single | Unknown primary |
| W A Hall | 2000 | 1973-1993 | Retrospective | 740 | Multimodal | Single | Various |
| Boram Ha | 2016 | 2001-2015 | Retrospective | 51 | MRI | Multi | Breast |
| James L Frazier | 2010 | 2003-2007 | Retrospective | 237 | MRI | Single | Various |
| Kazuhiro Ohtakara | 2012 | 2006-2009 | Retrospective | 131 | MRI | Single | Various |
| Penny K Sneed | 1999 | 1991-1997 | Retrospective | 215 | Multimodal | Single | Various |
| Alfredo Mussi | 1996 | 1975-1992 | Retrospective | 52 | NR | Single | NSCLC |
| Ashraf S. Mahmoud-Ahmed | 2002 | 1984-2000 | Retrospective | 116 | Multimodal | Single | Breast |
| Jaap D. Zindler | 2017 | 2002-2015 | Retrospective | 495 | MRI | Multi | NSCLC |
| Patrick Rodrigus | 2001 | 1993-1998 | Retrospective | 250 | Multimodal | Single | NSCLC |
| Rosaline Ma | 2017 | 2011-2016 | Retrospective | 19 | Multimodal | Single | Various |
| Sun Ha Paek | 2005 | 1999-2002 | Retrospective | 208 | Multimodal | Single | Various |

CT: Computed Tomography

GYN: Gynecologic

MRI: Magnetic Resonance Imaging

Multi: Multi-institutional

NR: Not Reported

NSCLC: Non-Small Cell Lung Cancer

RCC: Renal Cell Carcinoma
